# Magnitude and associated factors of acute kidney injury among preterm neonates admitted to public hospitals in Bahir Dar city, Ethiopia 2022: cross-sectional study

**DOI:** 10.1186/s12887-023-04147-2

**Published:** 2023-06-29

**Authors:** Sayih Mehari, Silenat Muluken, Asmare Getie, Amare Belachew

**Affiliations:** 1 College of Medicine and Health sciences, School of Nursing, Arbamich University, Arba Minch, Ethiopia; 2 College of Medicine and Health Sciences, Bahir Dar University, Bahir Dar, Ethiopia

**Keywords:** Acute kidney injury, Preterm, Magnitude, Bahir Dar, Ethiopia

## Abstract

**Background:**

Acute kidney injury is an independent risk factor for morbidity and mortality in critically ill neonates. Although the magnitude of preterm neonates is high and a major risk for acute kidney injury, there is a paucity of information regarding the magnitude and associated factors of acute kidney injury among preterm neonates in the study area. Therefore, the aim of this study was to assess magnitude and associated factors of acute kidney injury among preterm neonates admitted to public hospitals in Bahir Dar city, Ethiopia, 2022.

**Methods:**

An institutional-based cross-sectional study was conducted among 423 preterm neonates admitted to public hospitals in Bahir Dar city from May 27 to June 27, 2022. Data were entered into Epi Data Version 4.6.0.2 transferred to Statistical Package and Service Solution version 26 for analysis. Descriptive and inferential statistics were employed. A binary logistic regression analysis was done to identify factors associated with acute kidney injury. Model fitness was checked through Hosmer-Lemeshow goodness of fit test. Variables with a p-value < 0.05 were considered as statistically significant in the multiple binary logistic regression analysis.

**Result:**

Out of 423 eligible, 416 neonatal charts were reviewed with a response rate of 98.3%.This study revealed that the magnitude of acute kidney injury was 18.27% (95% CI = 15–22). Very low birth weight (AOR = 3.26; 95% CI = 1.18–9.05), perinatal asphyxia (AOR = 2.84; 95%CI = 1.55–5.19), dehydration (AOR = 2.30; 95%CI = 1.29–4.09), chest compression (AOR = 3.79; 95%CI = 1.97–7.13), and pregnancy-induced hypertension (AOR = 2.17; 95%CI = 1.20–3.93) were factors significantly associated with the development of neonatal acute kidney injury.

**Conclusion:**

Almost one in five admitted preterm neonates developed acute kidney injury. The odds of acute kidney injury were high among neonates who were very low birth weight, perinataly asphyxiated, dehydrated, recipients of chest compression, and born to pregnancy-induced hypertensive mothers. Therefore, clinicians have to be extremely cautious and actively monitor renal function in those neonatal population in order to detect and treat acute kidney injury as early as possible.

## Background

Acute kidney injury (AKI) is described as a sudden drop in glomerular filtration rate (GFR), resulting in the retention of urea and other nitrogenous waste products as well as a loss of fluid, electrolytes, and acid-base balance [1]. It commonly occurs in the neonatal intensive care unit, where it particularly affects preterm neonates. Neonates are more likely to develop AKI in the first few days after birth. This is because they are born with high renal vascular resistance, low GFR, high plasma renin activity, decreased intercortical perfusion, and inadequate sodium reabsorption in the proximal tubules [2, 3].

Although there has been a decrease in the morbidity and mortality of premature babies through improved premature care in recent decades, acute kidney injury is still a high global burden [4]. A multinational 24-center study called Acute Kidney Injury Epidemiology in Neonates (AWAKEN) has shown that 30% of neonates have developed AKI during their hospitalization in a critical care unit, among whom 66% were less than 36 weeks of gestation at birth [5]. In another systematic review study conducted from studies done globally, it has been estimated to occur in 8.4 to 63.3% of critically ill preterm neonates admitted to neonatal intensive care units [6]. In Ethiopia, a single study conducted among the total neonatal population at Black Lion Hospital has shown that 12.7% have developed AKI, of whom only 11.8% were preterms [7].As nephrogenesis is not completed until 34 weeks of gestation, most preterms have immature kidneys at birth, with more functional insufficiency of the glomeruli and tubules than mature neonates [8]. Moreover, preterms are exposed to interventions in the NICU that promote survival but are nephrotoxic, which predisposes them to AKI [9]. Low birth weight, prematurity, hypoxic ischemic encephalopathy, perinatal asphyxia, therapeutic hypothermia, and congenital heart disease are factors putting newborns at higher risk for developing AKI than the baseline neonatal population [10–13].

As demonstrated in the AWAKEN study, most critically ill neonates survive after AKI, with numerous long-term complications [5]. Acute kidney injury can lead to chronic kidney disease, and premature babies with AKI are at a higher risk of developing long-term kidney diseases [14]. As a result, methods for identifying neonates at risk of AKI and establishing steps to avoid the development of AKI are critical, given the association of AKI with increased hospital stay and mortality [15].

Neonatal AKI has been linked to increased mortality and length of stay in the hospital, raising the cost of care and posing a problem for nations with low resources [5]. Most low- and middle-income countries, including Ethiopia, have limited facilities and qualified health-care personnel to care for patients with kidney illnesses, especially those who require renal replacement treatment, making AKI a double burden [16].

Despite preterm neonates being at high risk for AKI and its high magnitude in Ethiopia [17, 18], there is a paucity of information on the magnitude and associated factors of acute kidney injury among preterm neonates in the study area. Furthermore, this study is consistent with the global kidney research agenda on neonatal AKI, emphasizing the importance of understanding the magnitude and risk factors of AKI in guiding efforts on diagnosis and management [19]. Therefore, this study was aimed to assess the magnitude and associated factors of neonatal AKI among preterm neonates admitted to public hospitals in Bahir Dar city, Ethiopia.

## Methods

### Study area, design, and period

A multi-center, an institutional-based cross-sectional study was conducted from May 27, 2022, to June 27, 2022, at NICUs of public hospitals in Bahir Dar, Ethiopia. There are three public hospitals in Bahir Dar city, namely, Tibebe Ghion specialized hospital, Felege Hiwot comprehensive specialized hospital, and Addis Alem primary hospital. All these hospitals are currently providing intensive care services for neonates in need.

### Sample size determination and sampling procedure

A single population proportion formula, considering its assumption, was used to calculate the sample size. Considering the proportion of AKI among preterm neonates to be 50% and adding an incomplete chart (non-response) of 10% to intial sample size, the final sample size was 423. Also the Sample size for second objective was calculated and was less than 423. All preterm neonates admitted to the NICU of public hospitals in Bahir Dar city, from May 30, 2020, to April 30, 2022, were included in this study. Those neonates who died or were discharged before 24 h of neonatal age were excluded. A computer-generated, simple random sampling technique was employed to recruit study participants.

### Operational definition

In this study, ***AKI*** is defined by a serum creatinine-based KDIGO criteria physician diagnosis (i.e., an absolute serum creatinine rise ≥ 0.3 mg/dL or SCr rise ≥ 1.5–1.9 × baseline SCr within 48 h).

Independent variables were measured based on a confirmed physician diagnosis. Accordingly, a variable (case) is confirmed if the patient file has provided the following evidence for each variable:

#### Hyperbilirubinemia

In this study, it was defined as yellowish discoloration of the skin on physical examination plus a total serum bilirubin level greater than 5 mg per dL.

#### Congestive heart failure

positive for clinical findings of CHF plus ECG imaging, and or those who have started medication for it.

#### Respiratory distress syndrome

was diagnosed based on the presence of two or more of the following signs in their chart: an abnormal respiratory rate (> 60breath/minute), expiratory grunting, nasal flaring, chest wall recessions, and cyanosis.

#### Hyperthermia

a core body temperature beyond 39 °C measured axillary.

#### Neonatal sepsis

There should be two or three clinical signs from the following list. (i.e., apnea, difficulty breathing, cyanosis; tachycardia or bradycardia; poor perfusion or shock; irritability, lethargy, hypotonia, seizures; abdominal distension, vomiting, food intolerance, gastric residue, hepatomegaly; unexplained jaundice; body temperature instability; petechiae or purpura) in their chart.

#### Perinatal asphyxia

at least one of the following characteristics: A 10-minute Apgar score ≤ 5, need for resuscitation > 10 min, and metabolic acidosis (pH ≤ 7.0 from in the umbilical artery (UA) have to be documented in their chart.

#### Congenital renal anomaly

evidence of an ultrasound or CT scan showing a defect in the kidney occurred at birth, given that other possible causes of renal defects like trauma have to be ruled out.

#### Prolonged rupture of membrane

PROM is diagnosed when the time between rupture of the membrane and delivery is greater than or equal to 18 h.

### Data collection tools, procedures, and quality control

Data was collected using a checklist adapted from tools that had previously been used in similar studies [6, 20–23]. Data was collected from May 27 to June 27, 2022, through medical chart review by four nurses working in the study setting, and supervision of data collection was done by one MSc nursing student. Data quality was maintained by using a carefully designed tool (checklist) for data collection. A one-day training about the techniques of chart review and data extraction were provided for data collectors. A pretest was done on 5% (n = 21) of study subjects at Debre Tabor comprehensive specialized hospital. Face validity for the checklist was determined by supervisors and other clinical experts.

### Data processing and analysis

The data was checked for accuracy and consistency. It was then coded and entered into Epi Data version 4.6.0.2 for cleaning before being exported to SPSS version 26 for analysis. Both descriptive and inferential analysis were done. Descriptive data for a categorical variable was presented through frequency and percent, while for continuous data that was normally distributed, the mean with standard deviation and for non-normally distributed data, median with interquartile range were used. A binary logistic regression analysis was used to determine factors associated with acute kidney injury. Enter-method regression analysis was done to build the model. First, a bivariable binary logistic regression analysis was performed to find a factor with a 95% confidence interval of P value ˂0.25. Multicollinearity was checked between predictors using the variance inflation factor (VIF) and was found to have no significant correlation at a variance inflation factor (VIF) less than five. Then, variables associated with bivariable binary logistic regression were subjected to a multiple binary logistic regression analysis to control confounding variables. Finally, those factors with a P-value ˂0.05 at a 95% confidence level were considered as predictors of acute kidney injury among preterm neonates admitted to neonatal intensive care unit. The odds ratio was used to examine the strength of the relationship between outcome and predictor factors. The Hosmer-Lemeshow goodness of fit test was used to check the model’s fitness, and was fitted at 0.931. Finally, the findings were presented in the form of text, tables, and figures.

## ResultS

### Demographic characteristics of the study participant

Among 423 eligible study participants, 416 neonatal charts were reviewed, with a response rate of 98.3%. Regarding the sex of the neonates, 220 (52.9%) were male. The median (interquartile range) age at admission was 0.875 (0.04–1.00) days. The median (IQR) of the neonate’s weight at birth was 2150 (1900–2450) grams. The mean (SD) of gestational age was 31.87 (2.688) weeks, of which 254 (61.1%) were 32 weeks and above. Most (392, or 94.2%) of the participants were born in health facilities, of which 172 (43.88%) were inborn. (Table 1)

**Table 1:** Demographic characteristics of preterm neonates admitted to public hospitals in Bahir Dar city, Ethiopia, 2022, (n = 416)

*Characteristics*	*Category*	*Frequency (%)*
Sex	Male	220(52.9)
	Female	196(47.1)
Age at admission(in days)	≤one	330(79.3)
	>one	86(20.7)
Gestational age (in weeks)	˂32	162(38.9)
	≥ 32	254(61.1)
Place of delivery	Hospital	288(69.2)
	Health center	104(25)
	Home	24(5.8)
Inborn delivery	Yes	172(41.3)
	No	244(58.7)
Mode of delivery	SVD^a^	303(72.8)
	Instrumental	77(18.5)
	Cesarean section	36(8.7)
APGAR^b^ score at 1st minute	4–6	270(64.9)
	7–10	146(35.1)
APGAR score at 5th minute	4–6	89(21.4)
	7–10	327(78.6)
Birth weight (in grams)	1000–1500	44(10.5)
	1500–2500	301(72.4)
	≥ 2500	71(17.1)

^a^ Spontaneous vaginal delivery

^b^ Appearance, pulse, grimace ,activity, respiration

### Magnitude of Acute kidney injury

**Fig. 1:**
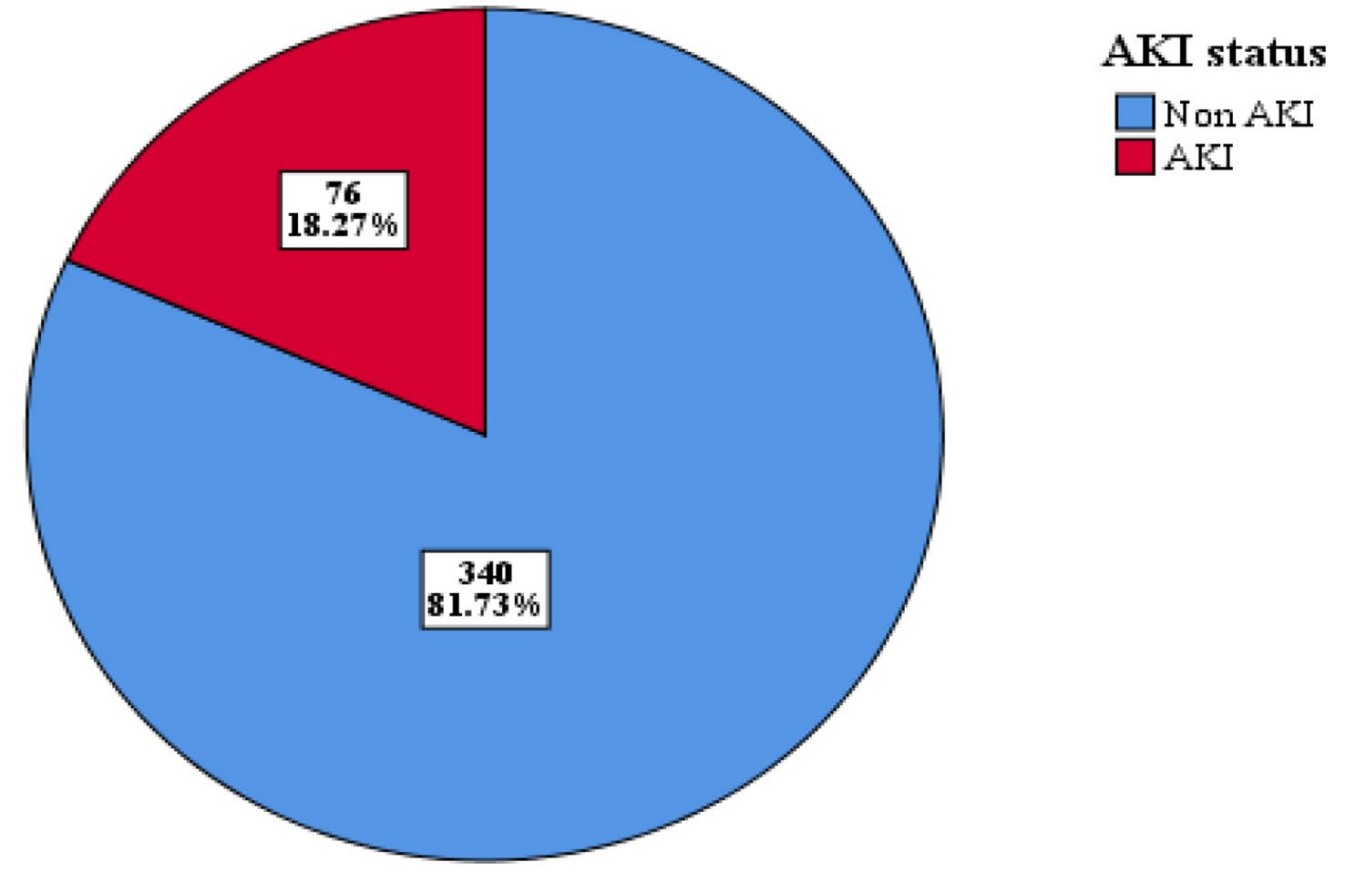
Magnitude of acute kidney injury among preterm neonates admitted to public hospitals in Bahir Dar city, 2022, (n = 416)

The magnitude of acute kidney injury among NICU-admitted preterm neonates in a public hospitals in Bahir Dar city was 18.27% (95% CI: 15, 22) (Fig. 1).

### Neonatal clinical factors

Out of 416 participants, 186 (44.7%) were septic, 156 (37.5%) were respiratory distressed, 160 (38.5%) were hyperbilirubinemic, 108 (26.2%) were perinataly asphyxiated, and 154 (37.5%) were dehydrated. More than half (224, or 53.8%) of the participants were provided with nephrotoxic drugs, of which 155 (69.1%) were aminoglycosides. Only 9 (2.2%) were diagnosed as having a congenital renal anomaly. Chest compression was done on 79 (19%) of the participants (Table 2).

**Table 2:** Other factor analysis of preterm neonates admitted to public hospitals in Bahir Dar city, Ethiopia, 2022, (n = 416)

	*Category*	*Frequency (%)*	*Variables*	*Category*	*Frequency (%)*
Perinatal asphyxia	Yes	108(26)	Congenital renal anomaly	Yes	9(2.2)
	No	308(74)		No	407(97.8)
Neonatal sepsis	Yes	186(44.7)	Diuretic drug use	Yes	65(15.6)
	No	230(55.3)		No	351(84.4)
Dehydration	Yes	154(37)	Chest compression	Yes	79(19)
	No	262(63)		No	337(81)
NEC^a^	Yes	85(20.4)	Radiant warmer use	Yes	123(29.6)
	No	331(79.6)		No	293(70.4)
CHD^b^	Yes	21(5)	Major surgery	Yes	16(3.8)
	No	395(95)		No	400(96.2)
Hyperthermia	Yes	29(7)	Incubation	Yes	119(28.6)
	No	387(93)		No	297(71.4)
RDS^c^	Yes	156(37.5)	Nephrotoxic drug use	Yes	224(53.8)
	No	260(62.5)		No	192(46.2)
Hyperbilirubinemia	Yes	160(38.5)	Nephrotoxic drug type (n = 224)	Aminoglycoside	155(69.2)
	No	256(61.5)		NSAID^d^	50(22.3)
				Others	19(8.5)
Pregnancy induced hypertension	Yes No	113(26.7) 303(73.3)	Prolonged rupture of membrane	Yes No	32(7.7)put them separetely384(92.3)
Number of neonate	Yes No	394(94.7) 22(5.3)	Chronic kidney disease	Yes No	52(12.5) 364(87.5)
Length of rupture of membrane(in Hours)	˂18 ≥ 18	384(92.3) 32(7.7)	Smoking	Yes No	11(2.6) 405(97.4)

^a^ Necrotizing enter colitis

^b^ Congenital heart disease

^c^ Respiratory distress disorder

^d^ Non-steroidal anti-inflammatory drug

### Maternal related charactestics

The mean (SD) of the mother’s age was 29.45(±7.75) years. The mean (standard deviation) time of membrane rupture was 2.54(±7.98) hours. Most mothers 394 (94.7%) gave birth to a single neonate from this neonate’s pregnancy. Only 11(2.6%) mothers had a smoking history while pregnant with their current neonate (Table 2).

### Factors associated with acute kidney injury

Among factors, birth weight, perinatal asphyxia, neonatal sepsis, neonate nephrotoxic drug use, dehydration, respiratory distress syndrome, chest compression, incubation, radiant warmer use, pregnancy induced hypertension, and prolonged rupture of membrane were associated with the development of neonatal AKI in bivariable binary logistic regression analysis at (p < 0.25).

Those variables that have an association with the outcome variable in bivariable binary logistic regression analysis were included in the multivariable binary logistic regression analysis. Birth weight, perinatal asphyxia, dehydration, chest compression, and pregnancy-induced hypertension during this neonate’s pregnancy were variables significantly associated with the development of neonatal acute kidney injury using multiple binary logistic regression at (p < 0.05).

Neonates with a birth weight of (1001–1500) grams were 3.26 times more likely to develop AKI than neonates with a birth weight greater than or equal to 2500 g (AOR = 3.26; 95% CI [1.18, 9.05]). In perinataly asphyxiated neonates, the odds of developing acute kidney injury were 2.84 times higher (AOR = 2.84; 95% CI [1.55, 5.19]) than in non-asphyxiated neonates. Neonates with a clinical diagnosis of dehydration were 2.30 times (AOR = 2.30; 95% CI [1.29, 4.09]) higher in terms of AKI development than non-dehydrated neonates.

Neonates who received chest compression were 3.74 times (AOR = 3.74; 95% CI [1.97, 7.13]) more likely to develop AKI than those who did not. Neonates from pregnancy-induced hypertensive mothers were 2.17 times (AOR = 2.17; 95% CI [1.29, 3.65]) more likely to develop AKI than neonates from non-hypertensive mothers (Table 3).

**Table 3:** Bivariable and multivariable binary logistic regression analysis for factors associated with acute kidney injury among preterm neonates admitted to public hospital in Bahir Dar city, 2022, (n = 416)

*Variable*		*AKI*		*Odds ratio*		*P-value*
		*Yes*	*No*	*COR(95%CI)*	*AOR (95%CI)*	
Birth weight	1001–1500	15	29	3.16(1.27–7.87)	3.26(1.18–9.05)	0.023*
	1501–2500	51	250	1.24(0.59–2.59)	1.17(0.52–2.65)	0.707
	≥ 2500	10	61	1	1	
Perinatal asphyxia	Yes	33	75	2.71(1.61–4.57)	2.84(1.55–5.19)	0.001*
	No	43	265	1	1	
Neonatal sepsis	Yes	42	144	1.68(1.02–2.77)	1.54(0.86–2.74)	0.145
	No	34	196	1	1	
Dehydration	Yes	40	114	2.20(1.33–3.64)	2.30(1.29–4.09)	0.005*
	No	36	226	1	1	
RDS	Yes	37	119	1.76(1.07–2.91)	1.56(0.88–2.74)	0.127
	No	39	221	1	1	
Nephrotoxic drug use	Yes	53	171	2.28(1.34–3.88)	1.76(0.97–3.19)	0.065
	No	23	169	1	1	
Chest compression	Yes	29	50	3.58(2.06–6.21)	3.74(1.97–7.13)	0.000*
	No	47	290	1	1	
Radiant warmer use	Yes	31	92	1.86(1.11–3.11)	1.50(0.82–2.76)	0.192
	No	45	248	1	1	
Incubation	Yes	30	89	1.84(1.09–3.09)	1.06(0.57–1.95)	0.86
	No	46	251	1	1	
PROM^a^	Yes	11	21	2.57(1.18–5.59)	2.01(0.79–5.16)	0.145
	No	65	319	1	1	
Pregnancy induced hypertension	Yes	31	82	2.17(1.29–3.65)	2.17(1.2–3.93)	0.010*

^a^ Prolonged rupture of membrane

## Discussion

The aim of this study was to determine the magnitude and associated factors of AKI among preterm neonates admitted to public hospitals in Bahir Dar city. In this study, the magnitude of AKI was 18.27% (95% CI: 15–22). This finding is comparable with studies done in Kenya (19.8%), Saudi Arabia (18.7%), Turkey (20.0%), and the AWAKEN study (18.8%) using the Neonatal KDIGO classification analysis of AKI by serum creatinine only [5, 7, 24, 25].

This finding is lower than studies done in the USA(38.0%) among extremely low gestational ages and (30.3%) among preterm neonates of less than 30 weeks of gestation [18, 26], Portugal (22.6%) among preterm neonates with ≤ 30 weeks of gestational age [27]. The possible explanation for this is a difference in the study design, study population gestational age, study setting (institutional level difference). Thus, in this study only 38.9% were less than 32 weeks of gestation. Due to prenatal fetal distress and exposure to numerous risk factor including infections, intrauterine growth retardation, placental insufficiency, and maternal medicine, extremely preterm newborns are more likely to develop AKI [28, 29].

This finding is also lower than studies done in Taiwan among extremely low birth weight neonates (56%), Serbia (26%), Egypt (44%), Saudi Arabia (56%), and Tanzania (31.5%) among neonates receiving care in level II and III NICUs [30–34]. The possible explanation for this difference may probably be attributed to the study population difference, a difference in study design, AKI definition, study setting, or study participant. Thus, extremely low-birth weight neonates have underdeveloped kidneys that can be easily affected by nephrotoxic drugs [35].

The finding of this study is higher than studies done at Tikur Anbesa Specialized Hospital(12.7%) [23], which could be attributed to a difference in neonatal gestational age, as only 11.8% were preterm in that study. Likewise, it is higher than a study done in Egypt (10.8%) in which 59.3% of the cases were preterm [36]. This could be due to the diagnosis of AKI in that study was defined by SCr greater than 1.5 mg/dl while in this study defined AKI an absolute serum creatinine rise ≥ 0.3 mg/dL or SCr rise ≥ 1.5–1.9 × baseline SCr within 48 h and there was also differences in included study populations. Also, this study finding is higher than studies conducted in northwest Parana state (7.5%), Iran (10.68%), India (12%), and United Arab Emirates (11.6%) [22, 37–39]. This may probably be due to study setting (institutional level) differences.

In line with previous research from Tikur Anbesa specialized hospital, Pakistan, and Iran [23, 40, 41], this study found perinatal asphyxia to be an independent predictor of AKI occurrence in NICU-admitted preterm neonates. Those perinataly asphyxiated preterm neonates were nearly three times more likely to develop AKI than non-perinataly asphyxiated. This could be because kidneys are extremely sensitive to oxygen deprivation, and as a result, renal insufficiency can occur within 24 h of a hypoxic ischemic episode, leading to irreversible cortical necrosis, if left untreated [12].

This study revealed that very low birth weight (1001–1500 g) was significantly associated with an increased risk of AKI in preterm neonates. Thus, the odds of AKI development were nearly three times higher in very low birth weight neonates than in normal birth weight (≥ 2500 g) neonates. This is consistent with a study conducted in Iran and a systematic review and meta-analysis done on risk factors for acute kidney injury [6, 22]. This might be attributed to the fact that low birth weight has contributed to the lower number and immaturity of nephrons, putting them at risk for AKI [42].

Chest compression was found to be an independent predictor of AKI occurrence in this study, with chest compression recipients being four times more likely to develop AKI than their counterparts. This finding is supported by a study conducted among asphyxiated neonates treated with therapeutic hypothermia [43]. This might be due to systemic ischemia/reperfusion injury due to the return of spontaneous circulation, thereby leading to multiple organ dysfunction syndrome (i.e., post-resuscitation syndrome), in which acute kidney injury (AKI) is one of the features of post-resuscitation syndrome [44, 45].

Dehydration was identified as a significant associated predictor of AKI, and dehydrated neonates were nearly two times more likely to develop AKI than non-dehydrated neonates. This finding is in line with a study done at Tikur Anbesa that has shown treatment for dehydration has an association with the occurrence of AKI [23]. This could be due to dehydration cause reduction in blood flow to the kidneys. Without blood flow providing oxygen to the kidneys, the kidneys do not work as well, causing prerenal damage.

Pregnancy-induced hypertension was identified as a significant predictor of AKI. Thus, neonates from pregnancy induced hypertensive mothers were nearly two times more likely to develop AKI than neonates to non-pregnancy-induced hypertensive mothers. This might be done by increasing the risk of premature birth and low birth weight [46]. This is in agreement with a study conducted in Turkey [20], while contradicting a study conducted in Taiwan which showed pregnancy induced hypertension as a protective factor against neonatal AKI [30].

### Limitation of the study

Since all hospitals used SCr based criteria of the neonatal modified KDIGO definition to diagnose AKI, a UoP based (oligouric) AKI was missed, which could underestimate the prevalence. And, due to the retrospective nature (use of secondary source) of the study, it presented limited predictors, although the predisposing factors for AKI are much broader.

## Conclusion

The magnitude of acute kidney injury among preterm neonates was high. Very low birth weight, perinatal asphyxia, dehydration, chest compression, and pregnancy induced hypertension were factors found to be independent predictors of acute kidney injury among NICU admitted preterm neonates. Therefore, clinicians have to be vigilant and actively monitor renal function in those preterm neonates to detect and manage AKI early. It is recommended to conduct further prospective follow up studies with larger sample size to address all possible predictors and accurately estimate its magnitude.

## Data Availability

The datasets used and/or analyzed during the current study are available from the corresponding author on reasonable request.

## List of Acronym

AKI: Acute Kidney Injury
AOR: Adjusted Odds Ratio
APGAR: Appearance Pulse Grimace Activity Respiration
AWAKEN: Acute Kidney Injury Epidemiology in Neonates
CHD: Congenital Heart Disease
CI: Confidence Interval
CKD: Chronic kidney disease
GFR: Glomerular Filtration Rate
KDIGO: Kidney Disease Improving Global Outcomes
NEC: Necrotizing Enter Colitis
NICU: Neonatal Intensive Care Unit
RRT: Renal Replacement Therapy
SCr: Serum Creatinine
